# Supplementary material for: Channel Capacity for Time-Resolved Effective Connectivity in Functional Neuroimaging
Source: bioRxiv. 2026 Mar 31:2026.03.28.714906. Preprint. [Version 1] doi: 10.64898/2026.03.28.714906 (PMC13060110; doi:10.64898/2026.03.28.714906)
Supplement: Supplement 1 [file media-1.pdf]

Supplementary Materials

(LF)

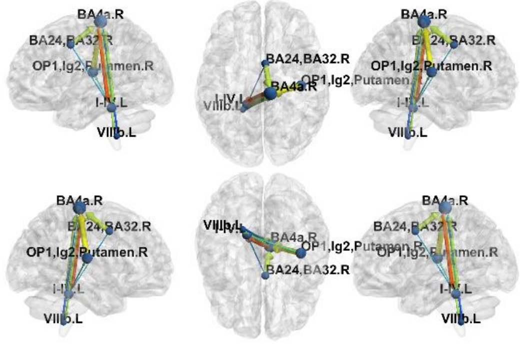

(RF)

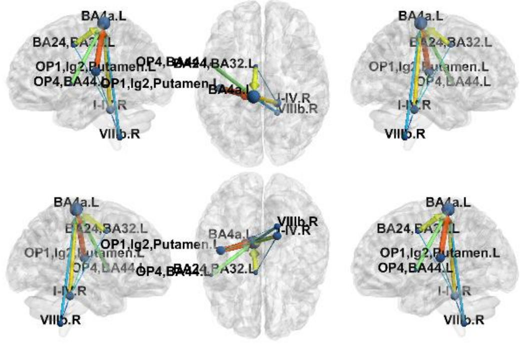

(LH)

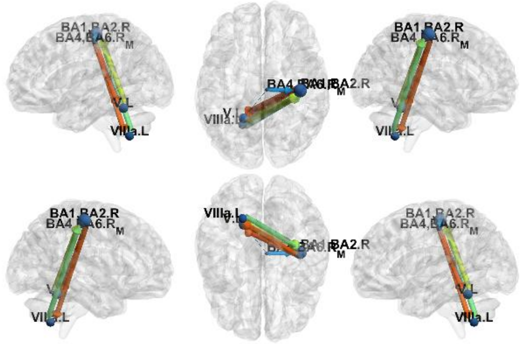

(RH)

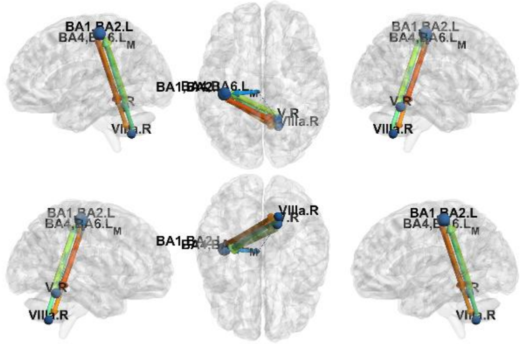

(T)

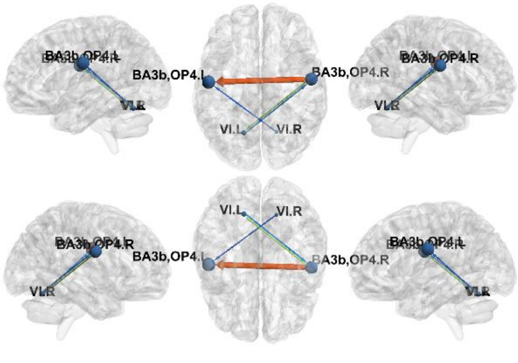

Fig. S1 ***Sagittal, axial, and coronal views of the motion-evoked connections.*** Directed connectivity in each movement type (LF, left foot; RF, right foot; LH, left hand; RH, right hand; T, tongue) was identified among task-specific ROIs using channel capacity at the 98% confidence level. Arrow size reflects channel capacity and arrow color reflects p-corr strength.

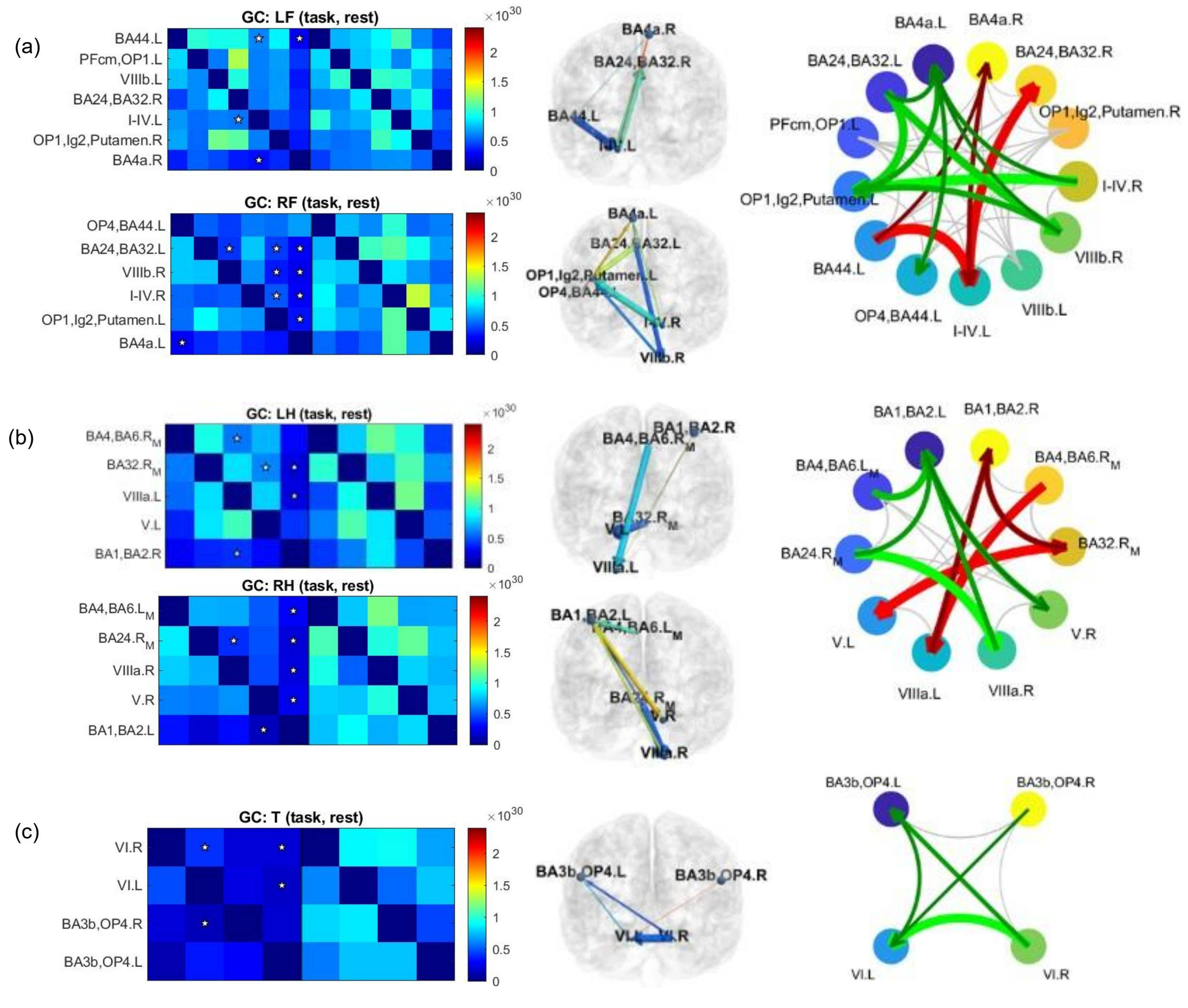

Fig. S2 Motion-evoked directed connectivity identified using Granger causality. Directed connectivity during foot movement (a), hand movement (b), and tongue movement (c) was identified among task-specific ROIs using channel capacity at the 76% confidence level. For each movement type, significant motion-evoked connections are marked with ☆ in the connectivity matrices (left), rendered on the brain cortex using BrainNet Viewer ((Xia et al., 2013); middle), and summarized as connectograms (right). In the cortical renderings, arrow size reflects Granger causality and arrow color reflects p-corr strength. In (a) and (b), left- and right-sided motion-evoked connections are shown together in the connectograms, with left-sided connections in red and right-sided connections in green. In (c), tongue-evoked connections are shown in green.

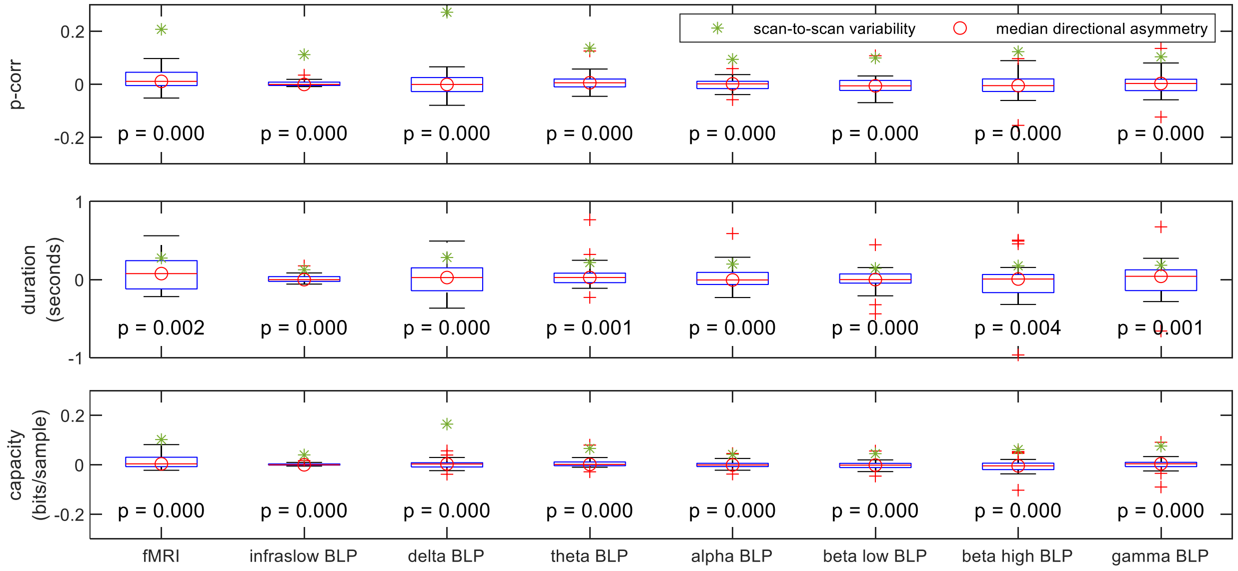

Fig. S3 Directional asymmetry of p-corr (top), duration (middle), and channel capacity (bottom) in the LFP-fMRI dataset. The p-values of the Wilcoxon signed-rank tests between directional asymmetry and scan-to-scan variability are shown below each boxplot.

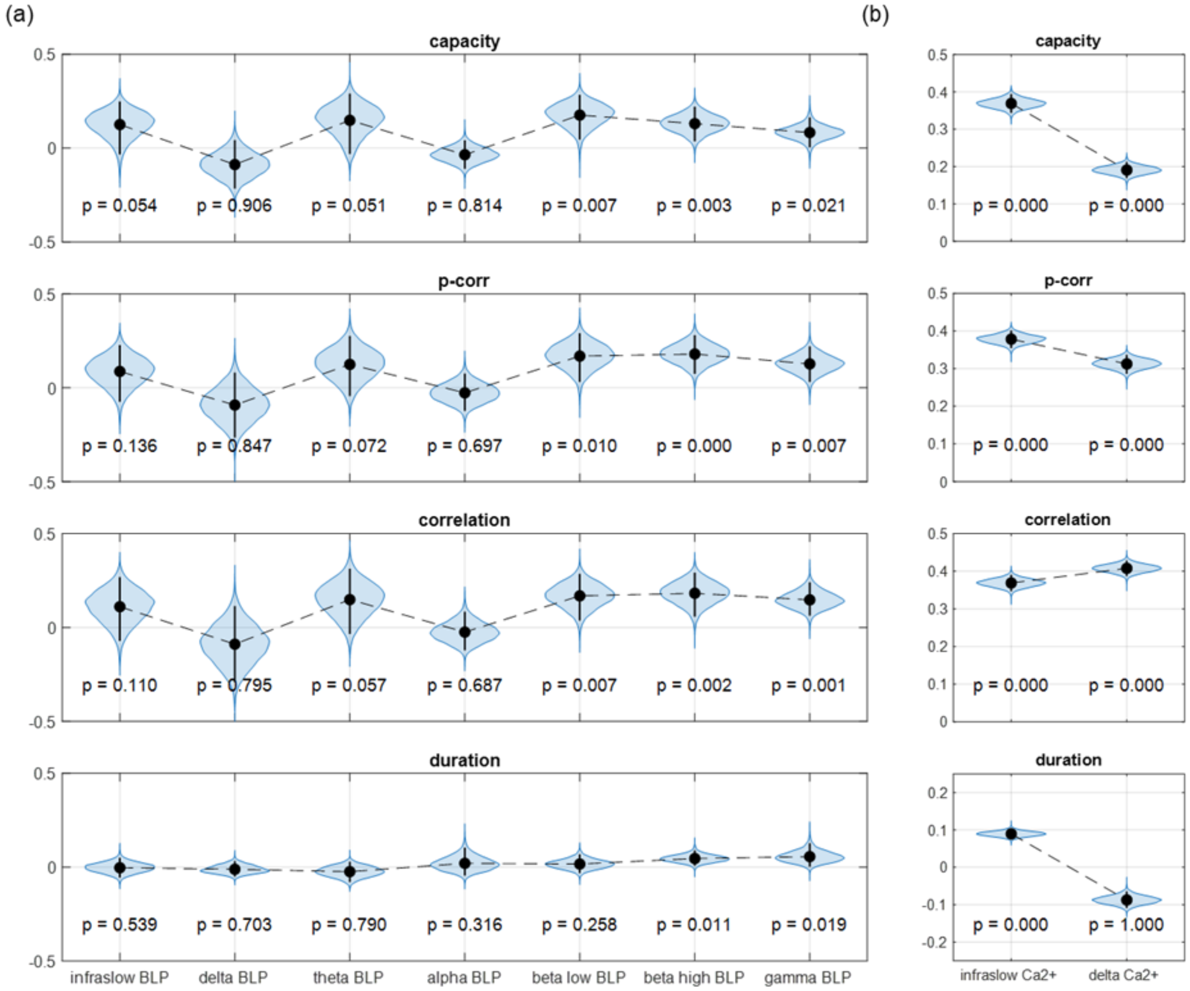

Fig. S4 Cross-modal comparison of BOLD-derived and neural (LFP/Ca2+-derived) connectivity measures (channel capacity, p-corr, correlation, duration). (a) Bootstrapped temporal correlations between concurrently acquired LFP- and BOLD-derived connectivity time series for each LFP band-limited power (BLP) band. (b) Ca^{2+}-fMRI results: bootstrapped spatial correlations between concurrently acquired Ca^{2+}- and BOLD-derived time-averaged connectivity matrices for the infraslow and delta components of the Ca^{2+} signals. P-values indicate whether the bootstrapped correlation is significantly greater than zero.

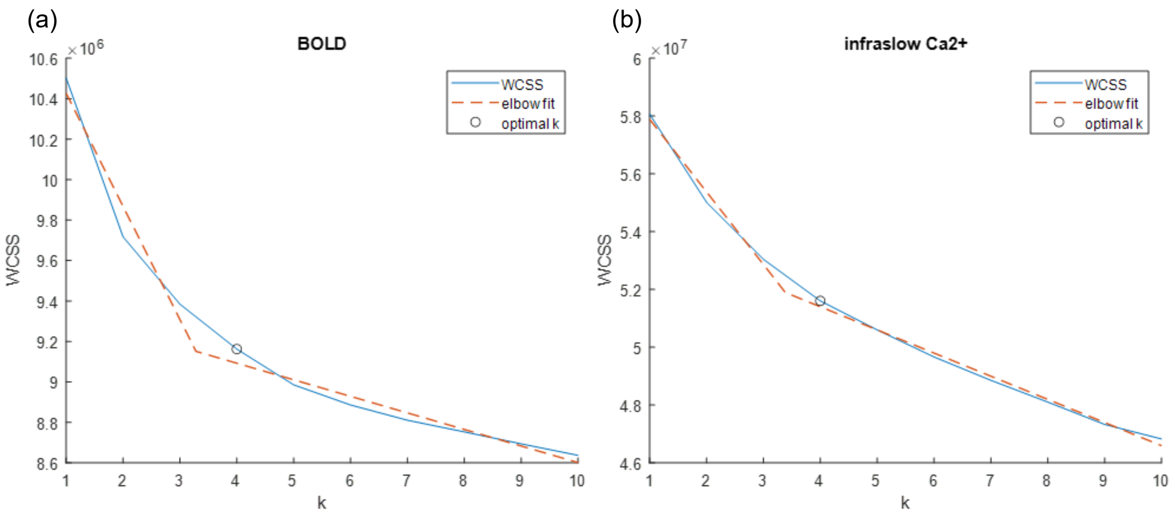

Fig. S5 Elbow method for determining the number of clusters in resting-state data, applied to (a) BOLD signals and (b) infraslow Ca^{2+} signals. The solid blue line shows the within-cluster sum of squares (WCSS) as a function of cluster number k, and the dashed red line indicates the two-segment linear fit. The optimal k was chosen as the smallest integer greater than the breakpoint.

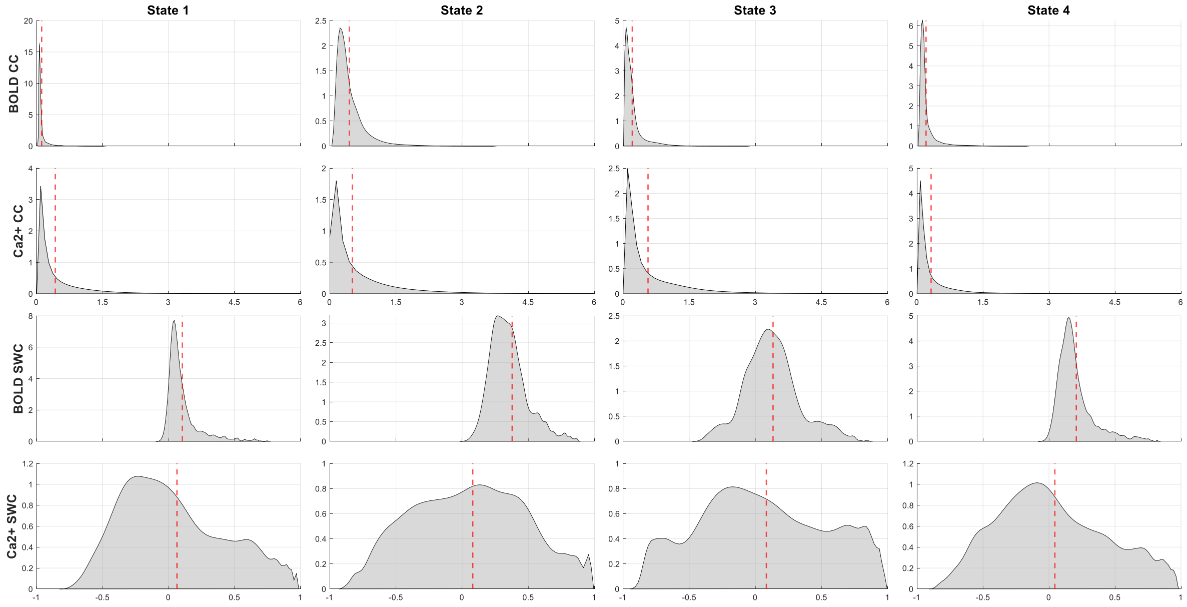

Fig. S6 Probability distributions of connectivity values in each of the four states. First row: channel capacity (CC) in the BOLD fMRI data. Second row: channel capacity (CC) in the infraslow band Ca^{2+} data. Third row: sliding-window correlation (SWC) in the BOLD fMRI data. Fourth row: sliding-window correlation (SWC) the infraslow band Ca^{2+} data. Dotted red lines mark the means.

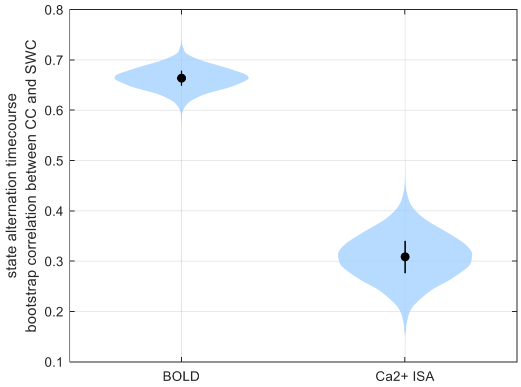

Fig. S7 Bootstrap correlation of the state alternation time course between channel capacity and SWC.

	State 1	State 2	State 3	State 4
fMRI centroids	0.90	0.96	0.94	0.97
Ca^{2+} centroids	0.64	0.67	0.71	0.78

Table S1. Correlation between channel capacity centroid and SWC centroid for each state.

	State 1	State 2	State 3	State 4
Capacity	0.56	0.54	0.53	0.65
SWC	0.35	0.44	0.37	0.60

Table S2. Correlation between fMRI centroid and Ca^{2+} centroid for each state using either Capacity or sliding-window correlation (SWC) connectivity measure.
